# A metabolomics study reveals potential plasma biomarkers for predicting post-infarction left ventricular remodeling: findings from the metabolights database

**DOI:** 10.3389/fcvm.2025.1723330

**Published:** 2025-12-17

**Authors:** Daqiu Chen, Yanqing Wu, Zhanxiong Xie, Jing Li, Shunxiang Luo, Shanghua Xu

**Affiliations:** Department of Cardiology, Nanping First Hospital Affiliated to Fujian Medical University, Nanping, Fujian, China

**Keywords:** biomarker discovery, left ventricular remodeling, plasma metabolites, ROC analysis, ST-segment elevation myocardial infarction, targeted metabolomics

## Abstract

**Background:**

Left ventricular remodeling (LVR) after ST-segment elevation myocardial infarction (STEMI) is a major determinant of adverse prognosis, yet reliable early biomarkers remain limited.

**Methods:**

This secondary analysis of publicly available metabolomic data from the MetaboLights database aimed to explore metabolic signatures associated with left ventricular remodeling (LVR) following STEMI patients. Key differential metabolites were identified through an integrated approach combining multivariate screening (PLS-DA with VIP >1.0) and univariate validation (Log2FC, FDR, Cohen's d). The robustness of principal findings was further verified by bootstrap resampling. These metabolites subsequently underwent comprehensive evaluation through univariate testing, ROC analysis, and Kyoto Encyclopedia of Genes and Genomes (KEGG) pathway enrichment.

**Results:**

Exploratory analysis revealed distinct differences in plasma metabolic profiles between patients with and without LVR. Notably, levels of threonine, glycine, and histidine were observed to be lower in the LVR group, with their respective AUCs of 0.785, 0.748, and 0.745 suggesting potential predictive value. Pathway analysis suggested an enrichment of these metabolites in processes such as amino acid metabolism and energy regulation, which might be linked to disruptions in oxidative stress, inflammatory, and fibrotic pathways.

**Conclusions:**

In summary, our preliminary findings suggest that threonine, glycine, and histidine may merit further investigation as potential biomarkers for post-STEMI LVR, and their association with key pathophysiological pathways hints at possible mechanistic roles. Given the absence of clinical and demographic data, combined with the small and imbalanced sample (6 LVR vs. 55 Non-LVR patients), our findings should be interpreted as exploratory and hypothesis-generating. Their clinical predictive utility requires validation in larger, independent prospective cohorts.

## 1 Introduction

Acute myocardial infarction (AMI), particularly ST-segment elevation myocardial infarction (STEMI), remains a leading cause of mortality and disability worldwide (1). While early reperfusion therapies, such as percutaneous coronary intervention (PCI), have significantly improved survival rates, a substantial proportion of survivors develop adverse left ventricular (LV) remodeling and subsequent heart failure (HF), which critically determine long-term prognosis (2, 3). The early identification of patients at high risk for post-infarction LVR remains an unmet clinical need, highlighting the demand for predictive biomarkers (4).

The pathophysiological process of LVR involves complex interactions among inflammation, fibrosis, oxidative stress, and notably, metabolic dysregulation (5–7). Metabolomics, which provides a dynamic snapshot of physiological and pathological states, has emerged as a promising approach for biomarker discovery in cardiovascular disease (8, 9). While previous metabolomic studies in AMI have identified biomarkers for diagnosis or general prognosis, the specific metabolic signatures predictive of LVR in the acute phase of STEMI remain poorly characterized (6, 10, 11).

This study therefore aimed to identify novel plasma metabolic biomarkers and dysregulated pathways specifically associated with post-STEMI LVR by performing a targeted metabolomic analysis, with the goal of improving early risk stratification.

## 2 Materials and methods

### 2.1 Data source and processing

The metabolomic data and associated clinical information were obtained from the MetaboLights database (12) (Study Identifier: MTBLS10634). This dataset comprises plasma samples and targeted metabolomic profiles from 61 patients diagnosed with ST-segment elevation myocardial infarction (STEMI). The diagnostic criteria for STEMI were defined as ST-segment elevation ≥2 mm in at least two contiguous peripheral or precordial leads on electrocardiogram (mean ST-segment elevation: 3.58 ± 1.96 mm). All enrolled STEMI patients received standardized acute-phase treatment, including intravenous thrombolysis (using alteplase or non-immunogenic streptokinase), followed by percutaneous coronary intervention (PCI) with stent implantation. In addition, patients received standard adjunctive therapy, including intravenous heparin (70 IU/kg), clopidogrel, and aspirin, as well as intravenous fentanyl, morphine, or nitroglycerin as needed for chest pain relief (13). This study was conducted at St. Joseph Clinical Hospital in the Belgorod region, Russia, involving patients with STEMI and ventricular remodeling. After a 12 h overnight fast, venous blood samples were collected in the morning using EDTA-containing tubes as anticoagulants at admission and on days 10, 30, and 90 post-admission. Samples were immediately centrifuged at 2,000 rpm for 20 min at 4 °C and then stored at −80 °C until metabolomic analysis. The metabolomic data were generated using absolute quantitative methods as described in the source literature (13). Specifically, the concentrations of amino acids and acylcarnitines were quantified using isotope-labelled standards from the MassChrom Non Derivatized 57,000 Kit (Chromsystems, Germany). Therefore, the metabolite levels reported in this dataset and used in our analysis are expressed in micromoles per liter (µmol/L).

#### 2.1.1 Study cohort and eligibility criteria

Based on the source literature (13), eligible participants were aged ≥18 years, diagnosed with STEMI or Canadian Cardiovascular Society (CCS) class III stable angina, and provided informed consent. Exclusions included Myocardial infarction without ST-elevation, CCS class I/II/IV angina, type 1 diabetes, use of specified supplements/medications prior to blood sampling, and any condition potentially confounding results or limiting participation. The study protocol was approved by the local ethics committee, and all participants provided written informed consent. The STEMI cohort (*n* = 61) had the following median [IQR] baseline values: blood glucose, 10.1 [7.4–10.9] mmol/L; creatinine, 75.5 [62.5–85.6] µmol/L; ALT, 51.0 [24.3–60.7] U/L; LDL-c, 3.52 [3.00–4.22] mmol/L.

### 2.2 Study groups and definitions

Based on subsequent echocardiographic assessments, patients were divided into two groups (13): the ventricular remodeling group (infarction_VR, *n* = 6), comprising patients who developed adverse left ventricular remodeling, and the non-remodeling group (infarction, *n* = 55), comprising patients who did not develop adverse left ventricular remodeling.

### 2.3 Metabolomic data processing and statistical analyses

#### 2.3.1 Data preprocessing

To correct for variations in the overall metabolite concentration profiles across different samples, median normalization was first applied to center the data and minimize systematic technical errors. Subsequently, a log_2_ transformation was performed to stabilize variance, approximate a normal distribution, and reduce the influence of extreme values. For multivariate statistical analyses, including partial least squares-discriminant analysis (PLS-DA), these preprocessed data were used. Specifically for the PLS-DA modeling, the data were further scaled to unit variance (scaleC = “standard”) to ensure equal weighting of all metabolites. The model's predictive performance was internally validated using five-fold cross-validation (crossvalI = 5). The detailed R code for data preprocessing is provided in the Supplementary Materials under the file names R_normalization.txt and Figure 2R.txt.

#### 2.3.2 Statistical analysis and metabolite screening

Given the sample size constraints and the risk of overfitting in multivariable models, we employed a sequential analytical strategy. We first performed multivariate analysis for preliminary screening, followed by univariate validation to robustly identify differential metabolites.

Preprocessed metabolite concentration matrices were downloaded from the MetaboLights database. All analyses were conducted using R version 4.4.1. Unsupervised principal component analysis (PCA) was initially applied to examine the overall data distribution (14). Subsequently, supervised partial least squares-discriminant analysis (PLS-DA) was used to maximize the separation between groups and identify metabolites contributing most to this discrimination (15), based on variable importance in projection (VIP) scores with a threshold of >1.0. The model's quality was assessed using R^2^X, R^2^Y, and Q^2^ values, and its significance was validated through permutation testing with 100 permutations.

The key metabolites identified by PLS-DA were then subjected to univariate analysis. Intergroup differences were assessed using the Wilcoxon rank-sum test, with *p*-values adjusted for multiple comparisons via the Benjamini-Hochberg false discovery rate (FDR). The effect size of the differences was quantified using Cohen's d, and the diagnostic potential of each metabolite was evaluated by receiver operating characteristic (ROC) curve analysis, reporting the area under the curve (AUC) with 95% confidence intervals. To further assess the robustness of key findings, bootstrap resampling (1,000 iterations) was performed to calculate the 95% confidence intervals for the Log2 fold change (Log2FC). Finally, metabolites with VIP > 1.0 were submitted to MetaboAnalyst 6.0 for KEGG pathway enrichment analysis. A *p*-value <0.05 was considered statistically significant.

## 3 Results

### 3.1 Study population and metabolomic profiling

A total of 61 patients with acute ST-segment elevation myocardial infarction (STEMI) were selected from the MetaboLights database (Study Identifier: MTBLS10634). Subsequent echocardiographic assessments identified six patients who developed left ventricular remodeling, whereas 55 patients did not (Supplementary 1). Targeted metabolomic analysis of plasma samples from patients with and without ventricular remodeling identified 70 metabolites (Supplementary 1). As a secondary analysis of a public database, this study could not access comprehensive baseline demographic information (e.g., age, sex) from the original cohort. Nevertheless, all patients were managed according to uniform STEMI diagnostic criteria and acute-phase treatment protocols, ensuring consistency in clinical management and minimizing potential confounding factors.

### 3.2 Principal component analysis reveals group separation

**Figure 1:**
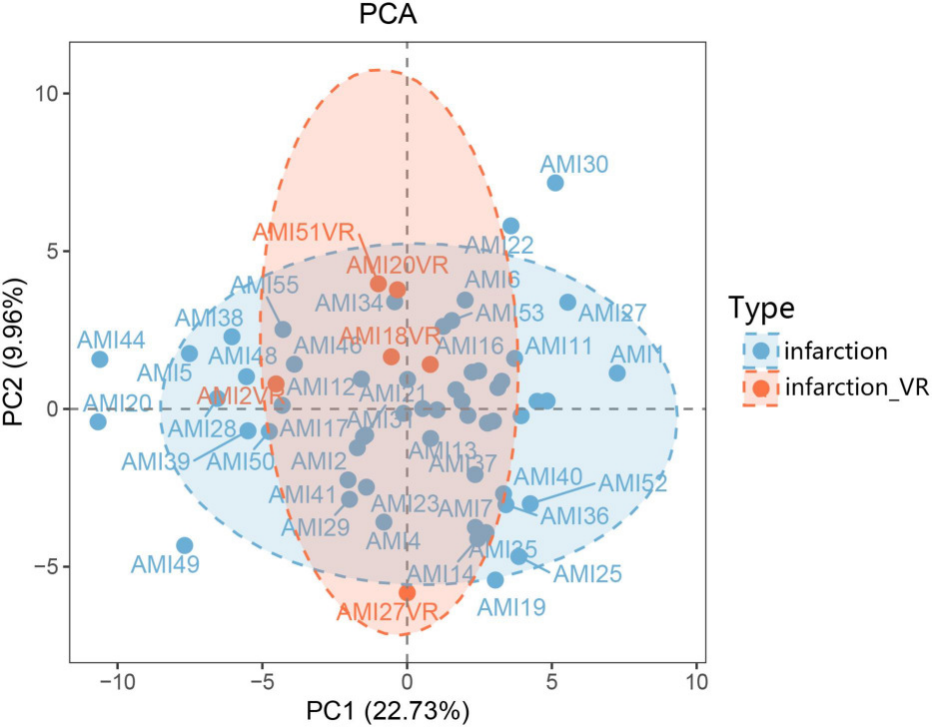
PCA score plot. The plot displays the distribution and separation of samples based on their multivariate profiles. The first two principal components, PC1 and PC2, explain 22.73% and 9.96% of the total variance, respectively. Data points are categorized by type: the non-remodeling group (infarction), and the ventricular remodeling group (infarction_VR). Individual sample identifiers are displayed for reference. PCA, principal component analysis.

Principal component analysis (PCA) was performed to visualize the overall metabolic distribution between patients with and without left ventricular remodeling. As shown in Figure 1, the two groups exhibited a partially separated distribution trend along PC1, which accounted for 22.73% of the total variance, whereas PC2 explained 9.96%. Although the infarction_VR group tended to cluster apart from the infarction group, some degree of overlap was observed, indicating moderate differences in overall metabolic patterns.

### 3.3 Statistical analysis and metabolite screening

**Figure 2:**
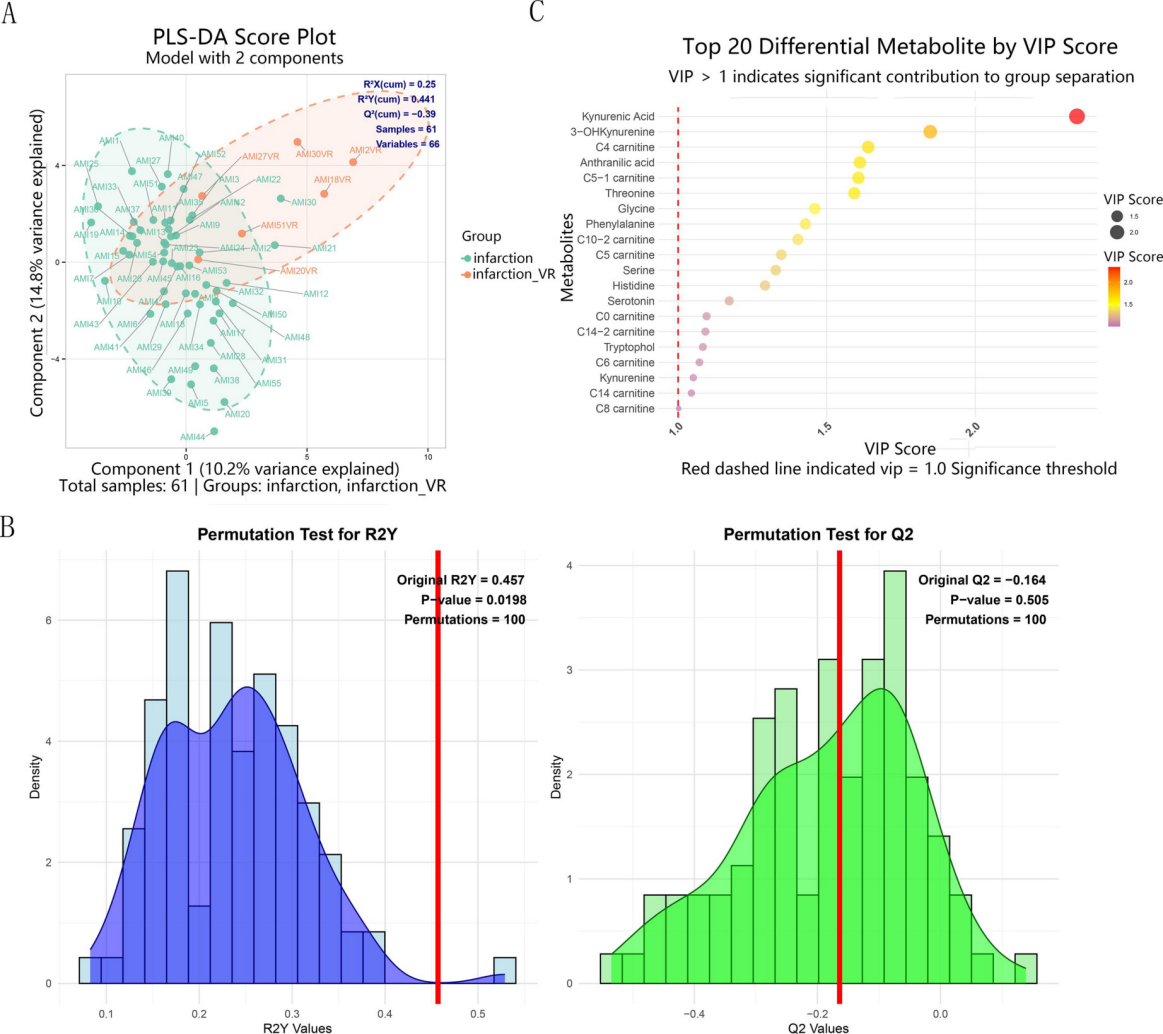
**(A)** PLS-DA score plot demonstrating separation between the non-remodeling (infarction) and ventricular remodeling (infarction_VR) groups. Key model parameters (goodness-of-fit: R^2^X, R^2^Y; predictive ability: Q^2^) are indicated. **(B)** Permutation test (*n* = 100) results for the PLS-DA model, showing the distributions of R^2^Y and Q^2^ from permuted data against the original model. **(C)** Distribution of VIP scores. PLS-DA, partial least squares-discriminant analysis; VIP, variable importance in projection.

PLS-DA analysis revealed a distinct separation trend in the metabolic profiles between the infarction and infarction_VR groups (R^2^Y = 0.441) (Figure 2A, Supplementary 2). Although intergroup differences were observed, the model exhibited limited predictive ability (Q^2^ = −0.39) (Figure 2A), which may be attributed to the limited sample size in the VR group (*n* = 6). VIP analysis identified 22 key metabolites with VIP values greater than 1.0, indicating their important contribution to group discrimination (Supplementary 3). The statistical significance of the PLS-DA model was assessed using permutation testing (*n* = 100 iterations, R^2^Y = 0.457, *Q*^2^ = −0.164) (Figure 2B). The permutation test results indicated that the separation trend between the two groups had a certain degree of explanatory power (*p* = 0.0198), while the predictive ability of the model was weak and did not reach statistical significance (*p* = 0.505). The top 20 metabolites ranked by VIP values were visualized using a bubble plot (Figure 2C, Supplementary 4).

To robustly identify differential metabolites between VR and Non-LVR patients post-AMI, we prioritized effect size (Cohen's d) alongside FDR. Metabolites with large effect sizes (|Cohen's d| > 0.8) were considered biologically relevant, even without a strict FDR < 0.05, prompting our focus on Threonine, Histidine, and Glycine. These were significantly downregulated in the VR group (Log_2_FC < 0, *p* < 0.01) with substantial effect sizes (Cohen's d = −0.96, −0.75, and −0.76, respectively). Importantly, bootstrap resampling confirmed the robustness of these findings, with 95% confidence intervals that did not cross zero for all three key metabolites. The top 20 differential metabolites between the Infarction_LVR and Infarction_Non-LVR groups, including all relevant statistical metrics, are presented in Table 1.

**Table 1:** Differential metabolites identified by PLS-DA and univariate analysis between ventricular remodeling (LVR, *n* = 6) and non-ventricular remodeling (Non-LVR, *n* = 55) groups.

Metabolite	VIP score	Non-LVR	LVR	Log_2_FC	*P*-value	FDR	Cohen's d	Bootstrap 95% CI
		Mean ± SD (µmol/L)	Mean ± SD (µmol/L)					
Threonine*	1.68	135.48 ± 38.72	99.53 ± 21.32	−0.45	0.006	0.21	−0.96	0.42–1.53
Histidine*	1.36	58.92 ± 17.29	46.36 ± 7.33	−0.35	0.006	0.21	−0.75	0.32–1.20
Glycine*	1.44	196.5 ± 53.19	156.8 ± 34.47	−0.33	0.037	0.85	−0.76	0.20–1.35
Methionine*	1.09	22.79 ± 9.49	17.48 ± 5.22	−0.38	0.061	0.85	−0.58	0.12–1.08
C0 carnitine	1.11	55.13 ± 16.25	45.49 ± 12.33	−0.28	0.122	0.91	−0.6	−0.004 to 1.26
3-OHKynurenine	1.76	0.79 ± 0.49	1.29 ± 0.66	0.71	0.125	0.91	0.98	−2.86 to 0.06
C5 carnitine	1.27	0.17 ± 0.12	0.25 ± 0.12	0.59	0.149	0.91	0.7	−2.14 to 0.09
C5-1 carnitine	1.61	0.02 ± 0.01	0.02 ± 0.01	0.47	0.147	0.91	0.89	−2.19 to 0.09
Kynurenic Acid	2.38	0.05 ± 0.01	0.07 ± 0.04	0.6	0.162	0.91	1.38	−3.09 to 0.07
C14 carnitine	1.11	0.04 ± 0.02	0.05 ± 0.01	0.31	0.174	0.91	0.48	−1.26 to 0.05
C4 carnitine	1.92	0.14 ± 0.13	0.36 ± 0.56	1.38	0.37	0.91	1.09	−2.84 to 0.54
Anthranilic acid	1.60	0.02 ± 0.02	0.04 ± 0.04	0.87	0.23	0.91	0.86	−2.15 to 0.08
Phenylalanine	1.42	80.26 ± 16.15	92.79 ± 22.98	0.21	0.24	0.91	0.74	−2.08 to 0.20
Kynurenine	1.18	0.82 ± 0.21	0.96 ± 0.36	1.17	0.23	0.91	0.63	−1.94 to 0.63
Serine	1.16	47.89 ± 13.21	42.28 ± 18.5	−0.18	0.5	0.91	−0.41	−0.56 to 1.63
C14-2 carnitine	1.12	0.08 ± 0.05	0.07 ± 0.02	−0.1	0.66	0.91	−0.11	−0.44 to 0.47
Tryptophol	1.11	0.01 ± 0.01	0.02 ± 0.03	0.87	0.49	0.91	0.57	−1.86 to 0.60
C6 carnitine	1.10	0.08 ± 0.05	0.08 ± 0.02	−0.06	0.79	0.94	−0.07	−0.48 to 0.45
C8 carnitine	1.06	0.2 ± 0.13	0.19 ± 0.05	−0.09	0.65	0.91	−0.1	−0.39 to 0.46
C14-1 carnitine	1.00	0.14 ± 0.09	0.15 ± 0.04	0.1	0.64	0.91	0.11	−0.72 to 0.25

Metabolite levels are presented as mean ± SD in µmol/L. Data were obtained via absolute quantitative targeted metabolomics as per the source study (13).

* *p*-value <0.05.

### 3.4 Differential expression patterns of key metabolites between groups

**Figure 3:**
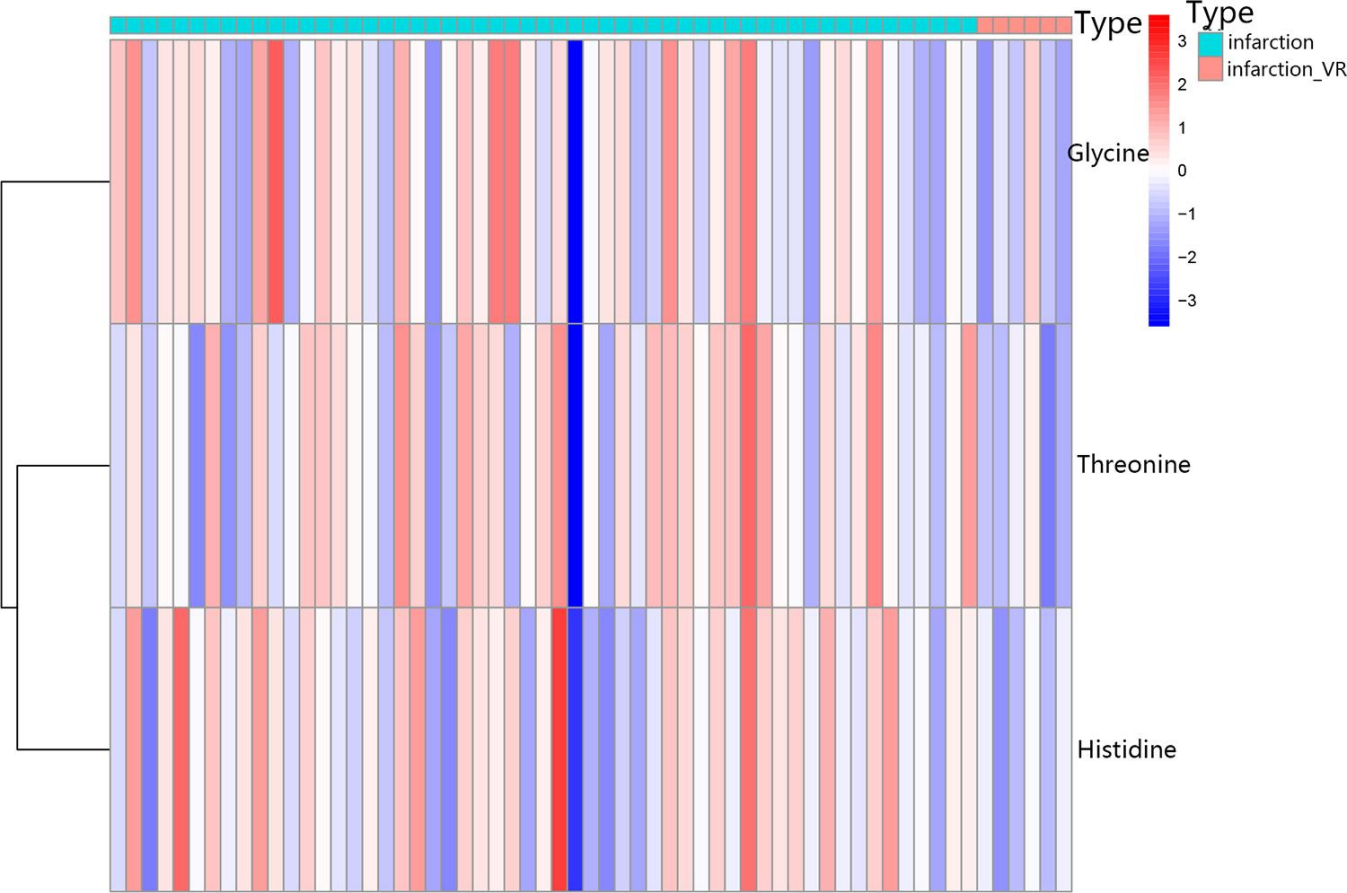
Heatmap of key differential metabolites. Levels of Threonine, Glycine, and Histidine across individual samples are displayed. Rows represent metabolites, and columns represent samples clustered by remodeling status (infarction vs. infarction_VR). The color scale indicates Z-score transformed levels, with red and blue representing concentrations above and below the mean, respectively.

To visualize the expression patterns of the key differential metabolites, a heatmap was generated for the top metabolites identified by the Variable Importance in Projection (VIP) analysis. As shown in Figure 3, the plasma metabolite profiles exhibited a clear clustering distinction between patients with ventricular remodeling (infarction_VR) and those without remodeling (infarction). Notably, the relative levels of threonine, glycine, and histidine were consistently and markedly lower in the infarction_VR group compared with the infarction group (Supplementary 5).

### 3.5 Validation of differential metabolite levels between groups

**Figure 4:**
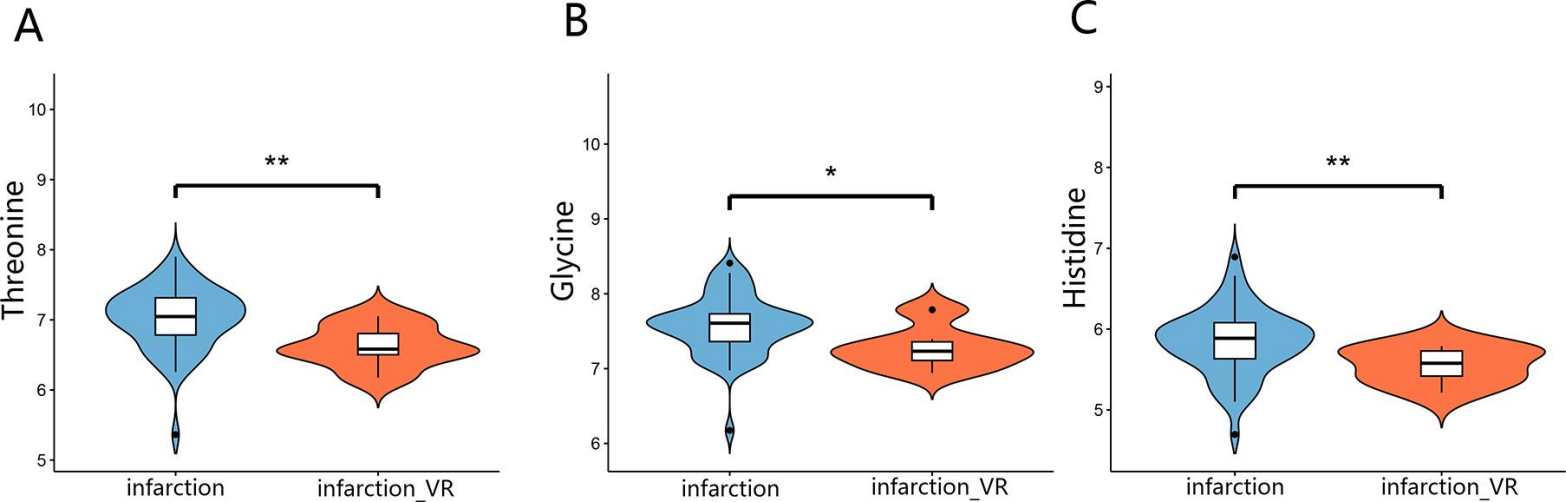
Violin plots of three key metabolite concentrations. Plasma concentrations of **(A)** Threonine, **(B)** Glycine, and **(C)** Histidine are compared between the non-remodeling (infarction) and ventricular remodeling (infarction_VR) groups. Each plot displays the data distribution, kernel density estimation, and median value. Statistical significance was determined by the Mann–Whitney *U* test (*p < 0.05, **p < 0.01).

To further confirm the differences in plasma concentrations of threonine, glycine, and histidine between the two groups, violin plots were generated (Figures 4A–C). As shown in the figure, patients who developed ventricular remodeling (infarction_VR group) exhibited significantly lower plasma levels of these metabolites compared with those without remodeling (infarction group). The shapes of the violin plots indicate that these differences were not driven by individual outliers but rather reflect a consistent downward shift in the overall distribution between the two groups.

### 3.6 ROC analysis of key metabolites

**Figure 5:**
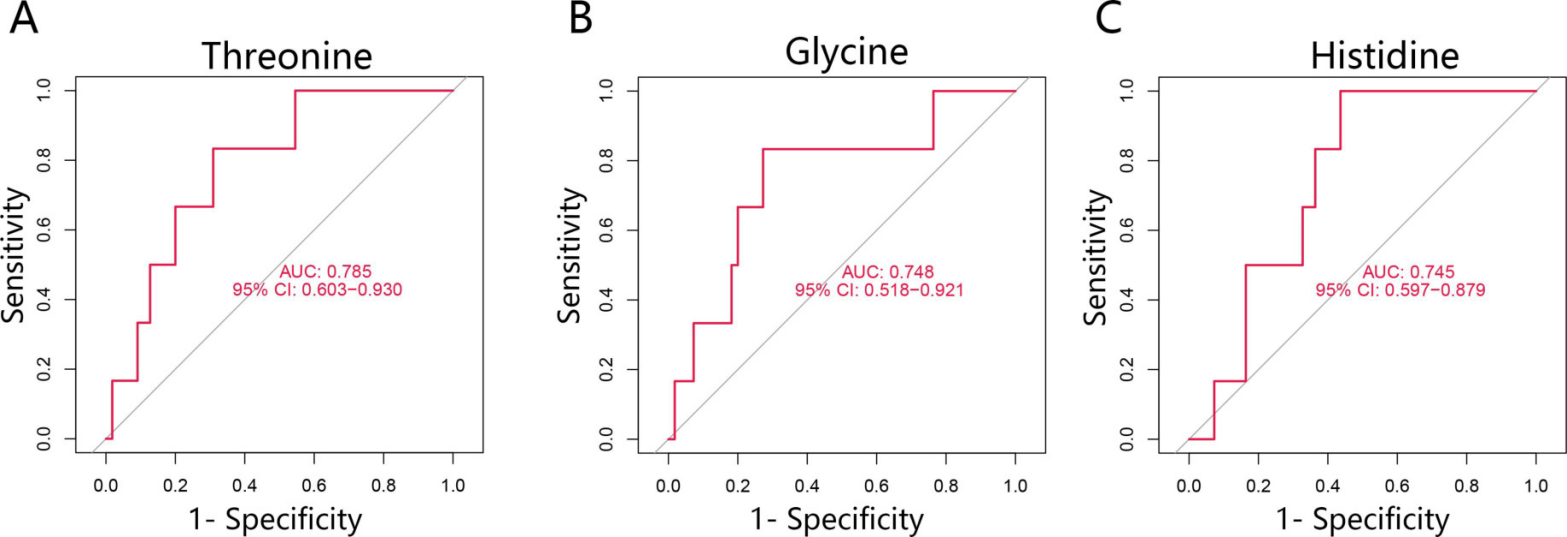
ROC curves of the three key metabolites. **(A)** The curves demonstrate the predictive performance of Threonine (AUC = 0.785), **(B)** Glycine (AUC = 0.748), and **(C)** Histidine (AUC = 0.745) for discriminating between post-STEMI patients with and without ventricular remodeling. ROC, receiver operating characteristic; AUC, area under the curve.

To assess the predictive value of threonine, glycine, and histidine for ventricular remodeling following ST-segment elevation myocardial infarction (STEMI), receiver operating characteristic (ROC) curve analysis was performed. As shown in Figures 5A–C, all three metabolites exhibited significant discriminative performance. Threonine showed the highest area under the curve (AUC = 0.785, 95% CI: 0.603–0.930), followed by glycine (AUC = 0.748, 95% CI: 0.518–0.921) and histidine (AUC = 0.745, 95% CI: 0.597–0.879). The lower bounds of the 95% confidence intervals for all three metabolites exceeded 0.5, confirming their statistically significant predictive capabilities.

### 3.7 Pathway enrichment analysis

**Figure 6:**
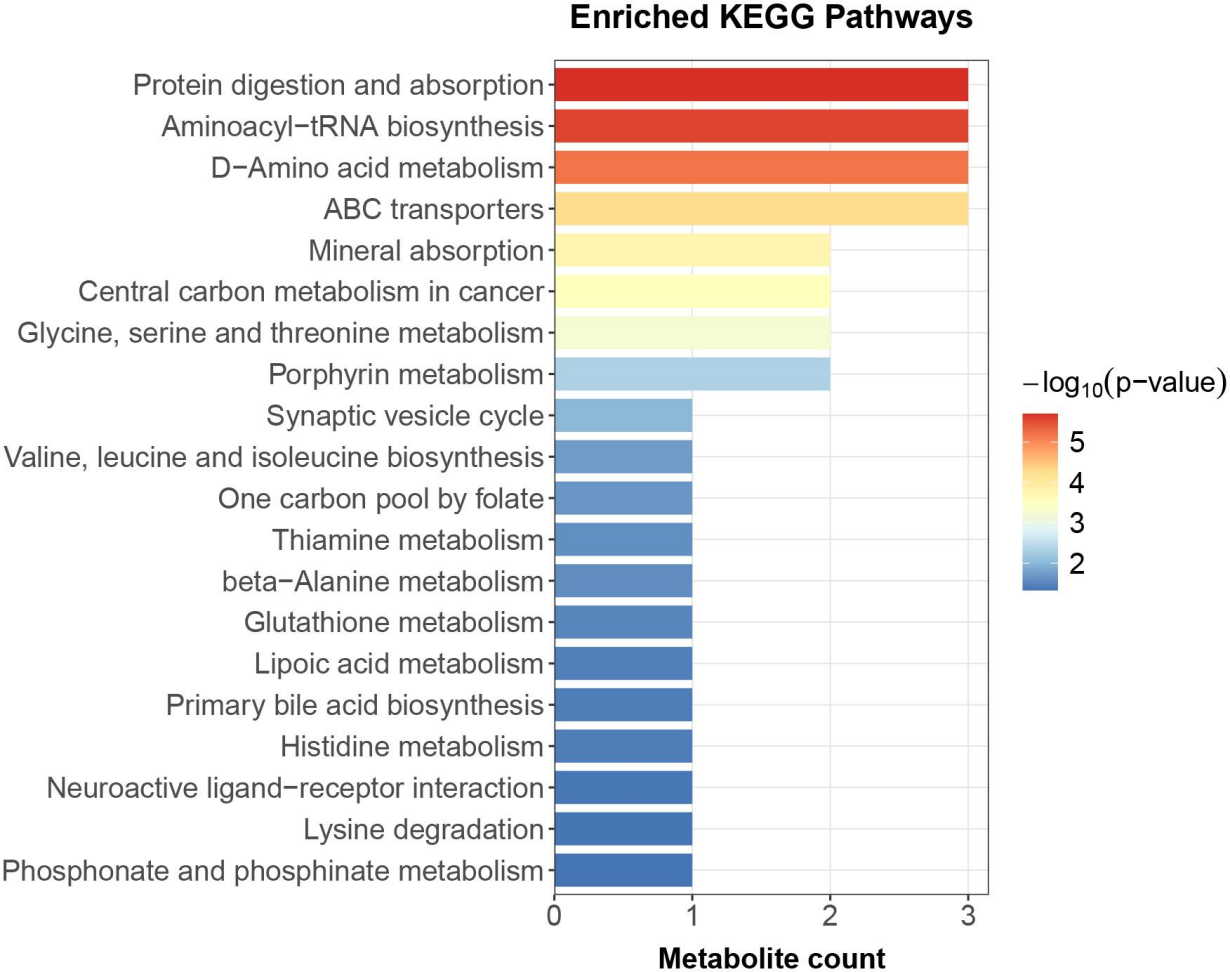
KEGG pathway enrichment analysis. The top 20 enriched pathways are shown. Bar length and color represent the −log10 (*p*-value) of enrichment. The number of metabolites mapped to each pathway is indicated. KEGG, Kyoto Encyclopedia of Genes and Genomes.

KEGG pathway enrichment analysis revealed that the differential metabolites were significantly enriched in 20 metabolic pathways. The most prominent pathways included Protein digestion and absorption, Aminoacyl-tRNA biosynthesis, D-Amino acid metabolism, ABC transporters, and Glycine, serine and threonine metabolism (Figure 6, Supplementary 6).

## 4 Discussion

This secondary analysis of publicly available targeted metabolomic data from the MetaboLights database investigated the relationship between early plasma metabolic profiles and left ventricular remodeling (LVR) after STEMI. While several previous metabolomic studies have examined broad prognostic markers in AMI (16), our study specifically focuses on predicting the distinct pathological process of LVR during the acute phase of STEMI. PCA indicated systematic differences in metabolic profiles between patients who developed LVR and those who did not. The pattern showed a tentative indication of group separation in the PLS-DA model; however, the model's limited predictive ability (Q^2^ = −0.39) likely reflects the modest sample size and class imbalance. Therefore, we prioritized univariate analyses—supplemented with Cohen's d and bootstrap 95% CIs—for more robust statistical evaluation.

We identified 22 metabolites with VIP scores >1.0, among which threonine, glycine, and histidine emerged as the most promising candidates. Univariate analysis indicated lower plasma levels of these metabolites in the LVR group compared to Non-LVR patients (Cohen's d = −0.96 for threonine, −0.76 for glycine, and −0.75 for histidine). These findings were further supported by consistent bootstrap confidence intervals. ROC analysis suggested these metabolites may serve as potential predictive biomarkers for LVR following STEMI.

The observed reductions in threonine, glycine, and histidine are mechanistically plausible within the context of LVR pathophysiology. Histidine serves as an important endogenous antioxidant that contributes to myocardial protection through multiple mechanisms. As a precursor of carnosine, it enables ROS scavenging and inhibition of lipid peroxidation, while concurrently enhancing the glutathione system and promoting nitric oxide synthesis to protect cardiomyocytes from oxidative injury (17, 18). Dysregulation of glycine and threonine metabolism is linked to inflammatory responses and fibrotic processes (19–23). Studies have demonstrated that glycine supplementation attenuates diabetic renal fibrosis, hinting at its potential antifibrotic role (24). Carnitine, whose synthesis depends on histidine, may suppress myocardial fibrosis by modulating calcium signaling and energy metabolism (22).

KEGG pathway enrichment analysis provided further mechanistic support, revealing significant enrichment of the “glycine, serine, and threonine metabolism” and “histidine metabolism” pathways. By focusing specifically on LVR, we uncovered a dysregulation in glycine, serine, threonine, and histidine metabolism, contrasting with the broader branched-chain amino acid (BCAA) changes associated with general prognosis (6), which may point to a more direct role in maladaptive remodeling. Concurrent enrichment of the “protein digestion and absorption” and “aminoacyl-tRNA biosynthesis” pathways suggests a systemic impairment in LVR patients, spanning from nutrient uptake to protein synthesis (25). This aligns with a state of negative nitrogen balance observed under cardiac overload and reflects the role of systemic energy metabolism dysregulation in driving ventricular remodeling (26, 27).

Building on associations between kynurenic acid and post-STEMI prognosis (16), our study provides novel evidence linking early depletion of threonine, glycine, and histidine specifically to left ventricular remodeling (LVR). The novelty of this re-analysis lies not in building a multivariate predictor—which our data cannot support—but in its rigorous, hypothesis-generating approach. Using a well-phenotyped public dataset and robust univariate validation, our analyses suggested specific, biologically plausible amino acid disturbances that may be linked to LVR. We hypothesize that this signature may reveal potential therapeutic targets. From a clinical-translational perspective, these key metabolites demonstrated promising predictive performance in ROC analysis. For instance, threonine achieved an AUC of 0.785, suggesting its potential utility as a component of a diagnostic panel for LVR risk. Future studies integrating these specific amino acids into a composite model, validated in larger prospective cohorts, could thus provide a more specific risk stratification tool than models derived from heterogeneous endpoints.

Several limitations of this study should be acknowledged. First, as a secondary analysis of a public database, this study lacked access to detailed clinical parameters, such as medication history and comorbidities. This precluded subgroup analyses and adjustment for potential confounders. Second, the sample size of the ventricular remodeling group was small (*n* = 6), resulting in significant class imbalance. While this imbalance likely contributed to the suboptimal performance of the PLS-DA model, the key metabolites—threonine, glycine, and histidine—showed consistent signals across multiple validation approaches. The observed association between these metabolites and LVR was supported by relatively large effect sizes (Cohen's d), stable bootstrap confidence intervals, and promising ROC performance, suggesting the potential robustness of these findings. Future studies with larger, balanced cohorts will be essential to confirm and extend these preliminary observations.

## 5 Conclusion

This study employed an integrated analytical strategy combining multivariate screening and univariate validation, which revealed significant downregulation of threonine, histidine, and glycine in the plasma of patients with post-STEMI ventricular remodeling. The substantial effect sizes and enrichment of relevant metabolic pathways suggest the potential involvement of these metabolites in the pathophysiology of ventricular remodeling. It must be emphasized that this work constitutes an exploratory analysis, and its findings are constrained by the limited sample size, group imbalance, and retrospective nature of the data; thus, all conclusions should be regarded as preliminary. Given these limitations, future studies involving larger, prospective cohorts are required to incorporate these metabolites into a systematic validation framework, with a focus on evaluating their predictive performance and translational potential when combined with clinical indicators, thereby providing more robust evidence for the early prediction of ventricular remodeling after STEMI.

## Data Availability

The datasets presented in this study can be found in online repositories. The names of the repository/repositories and accession number(s) can be found in the article/Supplementary Material.
